# Iodine nutritional status after the implementation of the new iodized salt concentration standard in Zhejiang Province, China

**DOI:** 10.1186/1471-2458-14-836

**Published:** 2014-08-12

**Authors:** Yan Zou, Xiaoming Lou, Gangqiang Ding, Zhe Mo, Wenming Zhu, Guangming Mao

**Affiliations:** Zhejiang Provincial Center for Disease Control and Prevention, 3399 Binsheng Road, Hangzhou, 310051 P.R. China

**Keywords:** Urinary iodine concentration, Comparison, Probability proportional to size sampling, Children

## Abstract

**Background:**

Iodine deficiencies were prevalent in China until the introduction of universal salt iodization (USI) in 1995. In 2012, the standard salt iodine concentration was adjusted to 20-30 mg/kg. The success of USI for the control of iodine deficiency disorders requires monitoring its effect at a population level.

**Methods:**

Two cross sectional surveys of a representative sample of children aged 8–10 years in Zhejiang Province were carried out in 2011 and 2013. Data on participants’ socio-demographic characteristics were collected from the children using a structured questionnaire. Spot urine samples were collected and delivered to local Center for Disease Control and Prevention laboratory for measuring urinary iodine concentration. In 2011, out of 420 selected children aged 8–10 years, 391 were recorded and provided urine samples. In 2013, out of 1560 selected children aged 8–10 years, 1556 were recorded and provided urine samples.

**Results:**

The median urinary iodine concentration of subjects in the 2013 survey was 174.3 μg/L, significantly lower than that of 2011(p = 0.000). The median urinary iodine concentration of subjects living in urban and rural areas in the 2013 survey was 169.0 μg/L, and 186.1 μg/L respectively, significantly lower than that of 2011 only for subjects living in urban areas (p = 0.000). There were no significant differences for subjects living in rural areas in the survey in 2011 and in 2013 (p = 0.086).

**Conclusions:**

At the time the new local iodization policy put forward, iodine nutrition was generally adequate in both urban and rural areas, suggesting that the new policy for adjusting the standard salt iodine concentration is effective. Our data also indicate that the reason people living in urban areas had a lower urinary iodine concentration than people in rural areas may be due to their preference for using non-iodized salt in the last 2 or 3 years. Maintaining USI at an appropriate level is an important part of preventing iodine deficiency disorders and should always be based on regular monitoring and comparison of urinary iodine concentration by province.

## Background

Over the past two decades, salt iodization has been introduced in many countries as a safe, cost-effective and sustainable way to eliminate iodine deficiency disorders (IDD) [1]. Iodine deficiencies were prevalent in China until the introduction of universal salt iodization (USI) in 1995. USI contributes significantly to the world’s disease and mortality burden [2, 3]. In the early 1980s, surveys identified 831000 individuals with IDD manifesting as goiter, and an additional 134 individuals with typical cretinism in Zhejiang Province. USI proved very effective; a provincial survey in 2000 found the virtual elimination of IDD. The success of USI for the control of IDD requires monitoring its effect at a population level. The principal indicator of effect is the median urinary iodine concentration, because it is highly sensitive to recent changes in iodine intake [4–6].

Median iodine concentration, measured in a spot urine sample from representative sample is the recommended method to assess the iodine nutritional status of a population [7]. Urinary iodine concentration could not provide direct information on thyroid function, but is a reliable measure of exposure, and a low median value suggests a population is at higher risk of developing thyroid disorders. Iodine deficiency is defined by the World Health Organization (WHO) as a population median urinary iodine concentration that falls below 100 μg/L [8]. Iodine nutritional status surveys are usually done in school age children because they are easy to reach through school based surveys and usually representative of the general population [9, 10].

In China, iodized salt is the main vehicle for iodine supplementation. Salt production is tightly controlled. There have been three adjustments on standard salt iodine concentration since 1995. In March 2000, the standard salt iodine concentration was 35 ± 15 mg/kg, and in 2012, the standard salt iodine concentration was adjusted to 20–30 mg/kg. This paper aims to compare the iodine nutritional status before and after the third adjustment of standard salt iodine concentration in children aged 8–10 years living in Zhejiang Province to provide reasonable suggestions to the government for policy making in the USI period.

## Methods

### Participants

Two cross-sectional surveys of a representative sample of Zhejiang Province in children aged 8–10 years were carried out in 2011 and 2013. Research protocols were approved by the Zhejiang Provincial Center for Disease Control and Prevention (CDC). Probability proportional to size sampling, in which the probability that a particular sampling unit would be selected in the sample is proportional to the population size of the sampling unit [11], was carried out in both surveys. With county as the sampling unit, 30 counties were selected from 89 counties. One primary school was then randomly selected from each county chosen. Finally schoolchildren aged 8–10 years from each school were selected.

### Measurement

Data on participant socio-demographic characteristics were collected from the children using a structured questionnaire. Spot urine samples were collected and delivered to a local Center for Disease Control and Prevention (CDC) laboratory for measuring urinary iodine concentration. Urinary iodine concentration was determined by the modified acid-digestion method [12].

### Ethics

This study was approved by the ethics committee of Zhejiang Provincial CDC. Informed written consent was obtained from parents or guardians of the children.

### Statistics

As continuous variables were not normally distributed, the results were described as median and range. The difference in urinary iodine concentration between 2011 and 2013 and the difference in urinary iodine concentration between rural areas and urban areas were evaluated by a nonparametric test (Mann–Whitney test), the differences in urinary iodine concentration among coastal, plain and mountain areas and among different age groups were evaluated by nonparametric test (Kruskal-Wallis H test). Data processing and statistical analyses were performed using SAS9.2 software (Cary, NC, USA). All tests were two-sided and the level of significance was set at p < 0.05.

## Results

In 2011, out of 420 selected children aged 8–10 years, 391 were recorded and provided urine samples (response rate = 93.1%). There were 196 males (50.1%) and 195 females (49.9%). The urban dwellers formed the biggest proportion of the children (72.1%); the rural dwellers constituted almost one-third (27.9%) of the children. In 2013, out of 1560 selected children aged 8–10 years, 1556 were recorded and provided urine samples (response rate = 99.7%). There were 733 males (47.1%) and 823 females (52.9%). The urban dwellers formed the biggest proportion of the children (62.1%); the rural dwellers constituted more than one-third (37.9%) of the children.

Table 1 presents the number of participants, median and range of urinary iodine concentration of subjects stratified by age in the 2011 and 2013 surveys. The median urinary iodine concentration of subjects in the 2013 survey was 174.3 μg/L, significantly lower than that of 2011 (p = 0.000). Furthermore, the median urinary iodine concentration of subjects aged 8, 9, and 10 in the 2013 survey were 173.0 μg/L, 181.5 μg/L, and 165.5 μg/L, respectively, also significantly lower than that of 2011 (p = 0.000). There were no significant differences among different ages in the survey of 2011 and 2013 (p > 0.05).

**Table 1:** **The sample size, median, and range of urinary iodine concentration of subjects in year 2011 and 2013 stratified by age**

	2011		2013		Z	*p*
Age	N	Median (range) (μg/L)	N	Median (range) (μg/L)		
8-year-old	102	245.3 (780.1)	489	173.0 (731.2)	−5.035	0.000
9-year-old	150	233.0 (761.2)	525	181.5 (936.3)	−4.990	0.000
10-year-old	139	243.7 (842.0)	542	165.5 (983.6)	−5.715	0.000
Total	391	237.1 (862.3)	1556	174.3 (586.0)	−9.119	0.000

Table 2 shows that salt iodine concentration of sampling site and the urinary iodine concentration of subjects in 2011 and 2013 stratified by 11 administrative divisions. Salt iodine concentration data were collected from the iodized salt survey carried out in 89 counties in 2011 and 2013. We present the median of iodized salt stratified by 11 administrative divisions as a reference value for the local situation. The median urinary iodine concentration of subjects in the 2013 survey was significantly lower than that of 2011 (p < 0.05).

**Table 2:** **The salt iodine concentration of sampling site and urinary iodine concentration of subjects in year 2011 and 2013 stratified by administrative divisions**

	2011		2013		Z	p
City	Salt Median (mg/kg)	Urinary iodine concentration Median (range) (μg/L)	Salt Median (mg/kg)	Urinary iodine concentration Median (range) (μg/L)		
Hangzhou	27.7	199.0 (648.1)	23.1	187.2 (936.5)	−0.665	0.506
Ningbo	27.0	254.9 (589.6)	23.3	154.8 (802.1)	−4.115	0.000
Wenzhou	27.8	232.6 (780.1)	23.9	151.8 (511.7)	−4.957	0.000
Jiaxing	27.0	205.5 (370.3)	23.9	175.9 (709.8)	−1.697	0.090
Huzhou	27.4	310.5 (233.0)	23.6	159.2 (488.6)	−4.111	0.000
Shaoxing	28.2	176.8 (396.0)	23.9	138.0 (324.0)	−2.321	0.020
Jinhua	29.2	243.0 (425.6)	24.6	192.2 (670.3)	−2.995	0.003
Quzhou	31.9	267.6 (479.3)	25.5	205.0 (488.5)	−1.808	0.071
Zhoushan	25.8	NR	24.0	170.6 (574.8)	NR	NR
Taizhou	28.2	298.2 (708.1)	24.3	210.2 (551.0)	−3.199	0.001
Lishui	31.7	452.2 (726.2)	26.1	243.7 (693.7)	−4.109	0.000
Total	30.5	237.1 (862.3)	24.1	174.3 (586.0)	−9.119	0.000

Table 3 presents the sample size, median, and range of urinary iodine concentration of subjects in 2011 and 2013 stratified by urban areas and rural areas in the 2011 and 2013 surveys. The median urinary iodine concentrations of subjects living in urban areas and rural areas in the 2013 survey were 169.0 μg/L and 186.1 μg/L, respectively, significantly lower than that of 2011 only for subjects living in urban areas (p = 0.000). There were no significant differences for subjects living in rural areas in the 2011 and 2013 surveys (p = 0.086). Furthermore, there were significant differences in the median urinary iodine concentration between subjects living in urban and rural areas both in the 2011 and 2013 surveys (p < 0.001). However the direction of the difference between urban and rural areas was opposite.

**Table 3:** **The sample size, median, and range of urinary iodine concentration of subjects in year 2011 and 2013 stratified by urban areas and rural areas**

	2011		2013		Z	*p*
Area	N	Median (range) (μg/L)	N	Median (range) (μg/L)		
Urban areas	282	254.8 (862.3)	967	169.0 (986.0)	−9.640	0.000
Rural areas	109	204.7 (545.0)	589	186.1 (596.2)	−1.719	0.086
Total	391	237.1 (862.3)	1556	174.3 (586.0)	−9.119	0.000

## Discussion and conclusions

China introduced a USI policy in 1995 with all edible salt (including table, food, and animal salt) iodized according to a national standard of 35 ± 15 from 2000. Yongning Wu et al. stated that salt contributed 63.5% of food iodine. Salt iodization assures iodine nutrition in China where environmental iodine is widely lacking [13]. In 2012, the Chinese government decided that while USI remains mandatory, provinces may iodize salt to a median within the range of 20–30 mg/kg. The reduction of iodized salt concentration underscores the need to monitor urinary iodine concentration carefully and regularly as an alternative and easy way of assessing iodine intake. This study was an extension of the preventative cross-sectional survey in Zhejiang Province in 2011 and provided a comparison of the difference in iodine nutritional status before and after the implementation of the new iodized salt concentration standard.

The urinary iodine concentration, when carried out with appropriate technology and sampling, is currently the most practical marker for iodine nutrition. Our results indicated that the median urinary iodine concentration of children aged 8–10 years was 174.3 μg/L in the 2013 survey, which was significantly lower than that of 2011 (237.1 μg/L). According to the criteria from WHO/UNICEF/ICCIDD 2007 for assessing iodine nutrition [14], the median urinary iodine concentration of children aged 8–10 years in 2011 was more than adequate (200–299 μg/L), suggesting that people might be at risk of iodine-induced hyperthyroidism. The iodine nutrition status of people was adequate after the adjustment in standard iodized salt concentration in 2012. The median urinary iodine concentration of children aged 8–10 years in the 2013 survey fell to 100–199 μg/L, suggesting an optimal iodine status and that the government’s decision to reduce the standard salt iodine concentration was a successful policy. In fact, China has made three adjustments since the implementation of USI. The adjustments were based on the iodine nutritional status of people by surveillance carried out every 2 years. Based on the recent surveillance results and risk assessment, the government decided to reduce the standard iodine concentration in 2012. Thus, maintaining USI at an appropriate level is an important part of preventing IDD and could have an important impact on maintaining people’s optimal iodine nutritional status.

The findings from this study also indicate that there were significant differences in median urinary iodine concentration between subjects living in urban and rural areas both in the 2011 and 2013 surveys. The direction of the difference between urban and rural areas were opposite, but the median urinary iodine concentration of the 2013 survey fell to 100–199 μg/L, suggesting an optimal iodine status in both urban areas and rural areas. Despite USI, non-iodized salt is still supplied in some markets. In rural or mountainous areas, with lower economic conditions compared with urban areas, people traditionally consume locally produced coarse salt and low-protein food, which resulted in a lower urinary iodine concentration when compared with people living in urban areas in the 2011 survey. This result was contrasted with previous studies to some extent [15, 16]. In recent years, some concerns about USI have circulated, for instance population trends in thyroid illness [17–19] and misconceptions about the risks posed by the diet of coastal residents, especially in urban areas. Urban residents believe they have sufficient iodine because they have more access to seafood as they live in coastal regions, and thus they prefer non-iodized salt [20, 21]. In addition, many local salt plants produce coarse salt, which results in consumption of non-iodized salt. Further knowledge, attitude, behavior and practice investigations in Zhejiang Province are essential to help in developing effective control measures and in monitoring their implementation. Our data from the 2013 survey indicate that people living in urban areas had a lower urinary iodine concentration than in rural areas, perhaps because of their preference for using non-iodized salt in the last 2 or 3 years.

Our study has some limitations. With spot collection of urine for measuring the urinary iodine concentration, the median of urinary iodine concentration is calculated to estimate the iodine status of a population, but it does not allow forming any conclusions of the iodine levels of a single individual [22, 23]. We present Table 2 of salt iodine concentration of sampling site and urinary iodine concentration of subjects in 2011 and 2013 stratified by 11 administrative divisions for reference. Limited by the sample size and measuring method, for every single administrative division, the median urinary iodine concentration of subjects could not represent the division level. But the table could provide related information of every sampling division to help us to learn broadly the changes before and after the implementation of the new iodized salt concentration standard.

This study has comprehensively compared the iodine nutritional status of a representative sample of 8–10-year-old children in two surveys carried out before and after the implementation of the new iodized salt concentration standard. Overall the median urinary iodine concentration declined between the surveys. At the time the new local iodization policy was implemented, iodine nutrition was generally adequate in both urban and rural areas, suggesting that the new policy for adjusting the standard salt iodine concentration is effective.. Maintaining USI at an appropriate level is an important part of preventing IDD and should always be based on regular monitoring and comparing urinary iodine concentration by province.
